# Quantifying cognitive resilience in Alzheimer’s Disease: The Alzheimer’s Disease Cognitive Resilience Score

**DOI:** 10.1371/journal.pone.0241707

**Published:** 2020-11-05

**Authors:** Tianyi Yao, Elizabeth Sweeney, John Nagorski, Joshua M. Shulman, Genevera I. Allen

**Affiliations:** 1 Department of Statistics, Rice University, Houston, TX, United States of America; 2 Department of Population Health Sciences, Weill Cornell Medicine, New York, NY, United States of America; 3 Departments of Neurology, Neuroscience, and Molecular and Human Genetics, Baylor College of Medicine, Houston, TX, United States of America; 4 Jan and Dan Duncan Neurological Research Institute, Baylor College of Medicine, Houston, TX, United States of America; 5 Departments of Electrical and Computer Engineering and Computer Science, Rice University, Houston, TX, United States of America; Nathan S Kline Institute, UNITED STATES

## Abstract

Even though there is a clear link between Alzheimer’s Disease (AD) related neuropathology and cognitive decline, numerous studies have observed that healthy cognition can exist in the presence of extensive AD pathology, a phenomenon sometimes called Cognitive Resilience (CR). To better understand and study CR, we develop the Alzheimer’s Disease Cognitive Resilience Score (AD-CR Score), which we define as the difference between the observed and expected cognition given the observed level of AD pathology. Unlike other definitions of CR, our AD-CR Score is a fully non-parametric, stand-alone, individual-level quantification of CR that is derived independently of other factors or proxy variables. Using data from two ongoing, longitudinal cohort studies of aging, the Religious Orders Study (ROS) and the Rush Memory and Aging Project (MAP), we validate our AD-CR Score by showing strong associations with known factors related to CR such as baseline and longitudinal cognition, non AD-related pathology, education, personality, APOE, parkinsonism, depression, and life activities. Even though the proposed AD-CR Score cannot be directly calculated during an individual’s lifetime because it uses postmortem pathology, we also develop a machine learning framework that achieves promising results in terms of predicting whether an individual will have an extremely high or low AD-CR Score using only measures available during the lifetime. Given this, our AD-CR Score can be used for further investigations into mechanisms of CR, and potentially for subject stratification prior to clinical trials of personalized therapies.

## Introduction

Alzheimer’s Disease (AD) is a debilitating, irreversible, and progressive brain disorder that destroys memory and cognitive skills. AD is a major public health concern, as it is the sixth leading cause of death in the United States and is the only cause of death in the top ten that cannot be prevented, cured, or slowed. A definitive diagnosis of AD requires the presence of AD pathology at autopsy (amyloid plaques and neurofibrillary tangles in the brain) in conjunction with cognitive symptoms observed during the lifetime [1]. Numerous studies show a link between AD-related pathology and cognitive decline, yet it has also been observed that healthy cognition can exist in the presence of extensive AD pathology [2]. In fact, only about 40% of the variation in late life cognition can be explain by pathology [3]. Consistently in community based cohorts with autopsy, around a third of people without dementia have the pathology meeting the criteria for intermediate or even high likelihood of AD [4–7]. A model for explaining this anomaly is the concept of cognitive resilience (CR). In [8], CR in AD is defined as the mechanism which enables some individuals to be more resilient to the pathological brain changes associated with AD than others; individuals with high CR show few or no clinical symptoms of AD during their lifetime, but have a pathological diagnosis of AD at autopsy. Understanding and characterizing CR could transform the way that AD is viewed from both a public health and scientific perspective, leading to population-level and targeted interventions as well as disease-modifying treatments and therapies to prevent, or slow progression of disease.

CR is an abstract concept with no consensus definition and therefore has been quantified using numerous approaches in the literature. Associations between levels of CR and a number of characteristics have been found in the literature, such as years of education, gender, social connectedness, and personality traits. One of the simplest and most frequently used methods to define CR is using one or more of these proxy variables [2, 9, 10]. In [11], a composite global enrichment score based on education, occupation and leisure activities is used as a surrogate for CR. Defining CR through the use of proxy variables has been cautioned against in the literature [12], as many of these proxy variables are highly correlated with other variables (such as socioeconomic status and health behaviors) already known to be associated with AD risk. In addition, many of the proxy variables do not reflect the same life experience among different individuals [9]. For example, one year of education could be very different among two different individuals.

CR has also been characterized using in-vivo structural magnetic resonance imaging (MRI) of the brain as a proxy for AD-related pathology. In [13], baseline episodic memory is decomposed into three components with a latent variable model: brain pathology (as characterized from measures derived from in-vivo structural MRI), demographic variables, and a latent person-specific factor that captures difference from the baseline test performance predicted for an average person with similar brain pathology and demographics. This latent factor defines CR and is extended to longitudinal settings in [14]. These methods rely heavily on parametric models, which may be prone to model misspecification, and do not produce stand-alone metrics of CR. And while measures derived from in-vivo structural MRI are available during the lifetime (unlike AD-related pathology measures), these measures have not been validated with ‘gold-standard’ postmortem pathology of AD and may not actually be related to brain pathology.

A number of studies have characterized AD-related CR by explaining the variation in cognition that remains after adjusting for AD-related postmortem pathology. In [15] and [16], CR is quantified by regressing a global cognition score on a global postmortem pathology score and an interaction between the pathology score and another characteristic (for example years of education). The interaction term tests the hypothesis that the characteristic modifies the effect of pathology on cognition, and is therefore associated with CR. In [17], a longitudinal global cognition score is modeled using a random effects mixture model, adjusting for common AD associated pathologies. After adjusting for these pathologies, four distinct groups of cognitive decline are found. Among the four groups, statistically significant differences are observed in depressive symptoms, measures of social isolation, and measures of cognitive and physical engagement activities. These residual-cognition methods have allowed for important discoveries of a number of important associations with CR, including experiential, psychological, and genetic findings [18, 19]. Yet, characterizing CR as the variation in cognition that remains after adjusting for AD-related pathology is not ideal, as these characterizations also use parametric models, which may be incorrectly specified, and do not produce stand-alone metrics of CR. Further, incorporating adjustment for AD pathologic burden in estimates of CR may obscure specific mechanisms conferring resilience to AD pathology, which may not necessarily be generalizable to other brain lesions. In addition, the characterizations using postmortem pathology are not available during an individual’s lifetime, and therefore cannot be used in clinical settings.

Here we propose an alternative framework for defining CR, the Alzheimer’s Disease Cognitive Resilience Score (AD-CR Score). Given AD-related pathology, the AD-CR Score is the difference between the observed and expected cognition. The AD-CR Score is closest to residual-cognition methods of characterizing CR, as it is computed using a global measure of cognition obtained proximal to death and ‘gold standard’ postmortem AD pathology. Yet unlike the residual-cognition methods, the AD-CR Score is fully non-parametric, stand-alone, and produces individual-level quantifications of CR that are derived independently of other measures. To show that the AD-CR Score is measuring CR, we validate the score by exploring its association with measures already known to be associated with CR, as well as longitudinal cognition. And while the AD-CR Score is computed using ‘gold-standard’ postmortem pathology of AD, we also propose a framework for predicting whether an individual will have an extremely high or low AD-CR Score using measures collected during premortem assessments, providing potential clinical utility to the score.

## Methods

### Participants

Data from two ongoing, longitudinal cohort studies of aging, the Religious Orders Study (ROS) and the Rush Memory and Aging Project (MAP) [18–20], is used for this analysis. The two studies were designed and are managed by the same team of investigators that collect the same measures, making the studies ideal to be combined for analysis. All participants are free of dementia when enrolled into the studies and agree to annual clinical evaluations and brain donation upon death. Both studies are approved by the Institutional Review Board of Rush University Medical Center.

At the time of this analysis, 3190 participants had completed the baseline evaluation and 1378 participants were deceased with completed brain autopsy. Clinical diagnoses were made following National Institute of Neurological and Communicative Disorders and Stroke-Alzheimer’s Disease and Related Disorders Association recommendations [21], and including AD, mild cognitive impairment (MCI), or no cognitive impairment (NCI). Such clinical diagnoses were made blind of all postmortem data. During autopsy, a modified NIA-Reagan score of AD pathology presenting as neurofibrillary tangles and neuritic plaques in the brain was also assigned [22]. This assessment is performed without knowledge of clinical information (e.g. a clinical diagnosis of dementia) and is a postmortem diagnosis of Alzheimer’s Disease based entirely on the neuropathology. Specifically, the modified NIA-Reagan score was dichotomized into low probability for having a pathologic diagnosis of AD and high probability for having a pathologic diagnosis of AD based entirely on the postmortem neuropathologic evaluation. Low probability means no or low likelihood of AD based on the postmortem neuropathologic evaluation. High probability means high or intermediate likelihood of AD based on the postmortem neuropathologic evaluation.

Of the 1378 participants who had died and gone to autopsy, 24 were excluded from this analysis because they were diagnosed with non-AD dementia at death, and 46 were missing either a clinical diagnosis at death or a modified NIA-Reagan score. Furthermore, the computation of our proposed AD-CR Score requires a global measure of cognition proximal to death and a global measure of AD pathology burden (described in the *Neuropsychological performance testing* section and the *Neuropathologic evaluation* section, respectively). Therefore, seven participants were excluded for not having the global AD pathology measure at autopsy. Similarly, we also excluded 319 participants who did not have the full battery of cognitive tests used to calculate the global cognition measure within two years of death. After applying this exclusion criteria, 980 subjects were used in the analysis.

We show a demographic summary for the 980 participants used in our analysis in Table 1 in terms of the premortem clinical diagnosis (i.e. AD, MCI, and NCI) as well as the postmortem pathologic diagnosis (i.e. the modified NIA-Reagan score). Nearly 15% of the participants have NCI from premortem clinical diagnosis with a high probability for having a pathologic diagnosis of AD based on postmortem neuropathologic evaluation. In other words, even though these participants actually have NCI before death, their postmortem neuropathologic evaluations show extensive AD-related pathology that would fulfill criteria for having a pathologic diagnosis of AD. These participants would be among those considered to have ‘high cognitive resilience (CR)’. In addition, over 18% of the participants have either MCI or AD from premortem clinical diagnosis despite a low probability for having a pathologic diagnosis of AD based on postmortem neuropathologic evaluation. These participants would be among those considered to be ‘cognitively vulnerable (CV)’. We want to note that Table 1 is only meant to provide a demographic summary for the participants and the actual definition/computation of our proposed AD-CR Score does not depend on the categorizations in Table 1 at all.

**Table 1:** A demographic summary for the n = 980 participants in terms of the premortem clinical diagnosis (i.e. AD, MCI, and NCI) as well as the postmortem pathologic diagnosis (i.e. the modified NIA-Reagan score).

		Clinical Diagnosis		
		AD	MCI	NCI
Pathologic Diagnosis	Low Probability of AD	66 (6.73%)	111 (11.33%)	212 (21.63%)
	High Probability of AD	286 (29.18%)	159 (16.22%)	146 (14.90%)

Of the participants, 146 (14.9%) had no cognitive impairment from premortem clinical diagnosis, yet a high probability for having a pathologic diagnosis of AD based on neuropathologic evaluation at autopsy. These participants would be among those considered to have ‘high cognitive resilience’.

### Neuropsychological performance testing

A battery of 21 cognitive tests are administered annually and nineteen of them are used to assess a variety of cognitive abilities across five cognitive domains (episodic memory, semantic memory, working memory, perceptual speed, and visuospatial ability) [20]. The specific tests used for each domain are summarized as follows: i) Logical Memory Ia, Logical Memory IIa, immediate story recall, delayed story recall, Word List Memory, Word List Recall, Word List Recognition for assessing episodic memory; ii) Boston Naming Test, Category Fluency (fruits, animals), National Adult Reading Test for assessing semantic memory; iii) Digit Span Forward, Digit Span Backward, Digit Ordering for assessing working memory; iv) Symbol Digit Modalities Test, Number Comparison, Stroop word reading, Stroop color naming for assessing perceptual speed; and v) Judgment of Line Orientation, Standard Progressive Matrices for assessing visuospatial ability [23]. A global measure of cognition is computed by converting each test to a z-score and then averaging the z-scores, as previously described in [24, 25]. Therefore, the global cognition measure is a summary measure of a subject’s overall cognitive abilities across five cognitive domains on a continuous scale—negative values for the global cognition measure indicate lower overall cognitive abilities than the average of the entire cohort whereas positive values signify higher cognitive abilities than the average of the cohort. This global cognition measure is used in the computation of our proposed AD-CR Score in our analysis.

### Neuropathologic evaluation

Following the procedures recommended by the National Alzheimer’s Disease Coordinating Center [26], the postmortem neuropathologic evaluation includes assessment of AD pathology, cerebral infarcts, lewy body disease, and other pathologies common in aging and dementia. In addition to the modified NIA-Reagan score, a global measure of AD pathology burden is constructed using three AD pathologies (neuritic plaques, diffuse plaques, and neurofibrillary tangles) from 5 regions of the brain (midfrontal cortex, midtemporal cortex, inferior parietal cortex, entorhinal cortex, and hippocampus), as previously described in [27]. This global measure of AD pathology burden is used in the computation of our proposed AD-CR Score in our analysis. As we are also interested in other, non-AD causes of dementia and cognitive decline, we accounted for the presence of Lewy bodies [28], the presence of hippocampal sclerosis [29], as well as measures of vascular infarcts: gross infarcts, microinfarcts, gross chronic infarcts, and chronic microinfarcts [30–33].

### Other measures

For this analysis, we investigate measures that may be correlated with CR and the AD-CR Score. We group these measures into the following categories to test association: demographic, cognition, non-AD pathology, education, personality, vision, APOE, pain, alcohol, smoking, BMI, comorbidity, parkinsonism, depression, life activities, and physical activities. The demographic group includes sex and age. The cognition group includes the summary measures of the five cognitive domains described in the *Neuropsychological performance testing* section (episodic memory, semantic memory, working memory, perceptual speed, and visuospatial ability). The non-AD pathology group consists of the non-AD pathology measures introduced in the *Neuropathologic evaluation* section, namely the presence of Lewy bodies, hippocampal sclerosis, gross infarcts, microinfarcts, gross chronic infarcts, and chronic microinfarcts. The education group is the number of years of education. The personality group includes of a measure of neuroticism [34] and a measure of anxiety [35, 36]. The vision group includes a test of visual acuity. The *APOE* genotype group includes an indicator of whether the individual has allele E2 as well as allele E4. The former has been shown to be protective for AD while the latter has been shown to increase an individual’s risk of AD [37, 38]. The pain group includes the measures of self-reported pain in the upper and lower extremities. The alcohol group includes self reported measures of the average grams of alcohol consumed per day in the last 12 months and the average number of alcoholic drinks consumed per day over the individual’s lifetime. The smoking group includes an indicator of being a former smoker or a current smoker. The BMI group is a measure of the individual’s BMI. The comorbidity group includes any history of hypertension, cancer, diabetes, head injury with loss of consciousness, thyroid disease, heart disease, and stroke. The parkinsonism group includes measures of bradykinesia, gait quality, rigidity and tremor all modified from the United Parkinson’s Disease Rating Scale (mUPDRS) [39] as well as an overall diagnosis of parkinsonism from a trained nurse based upon the mUPDRS scale [39]. The depression group is an assessment of depression using a modified Center for Epidemiologic Studies Depression (CESD) scale [40]. The life activities groups consists of a measure of the ability to perform instrumental activities of daily living [41], a measure of the ability to perform basic activities of living [42], and a measure of mobility disability [43]. The physical activities groups includes measures of general physical activity, swimming physical activity, walking physical activity, and physical activity spent doing yard work. Table 2 summarizes the measures in each of the groups.

**Table 2:** Summary of the measures that make up each of the groups for the analysis.

Group	Measure	Mean [Range]	Missing
Demographics	Age at Baseline	80.71 [62.64, 102.15]	0
	Male	37.65%	0
Cognition (z-scores, higher values for higher cognition)	Episodic Memory	-0.14 [-4.00, 1.45]	2
	Visuospatial Ability	-0.12 [-3.24, 1.61]	4
	Perceptual Speed	-0.17 [-3.18, 2.54]	3
	Semantic Memory	-0.07 [-5.73, 2.35]	2
	Working Memory	-0.05 [-3.39, 2.19]	1
Non-AD Pathology	Hippocampal Sclerosis	6.08%	9
	Lewy Bodies	19.71%	1
	Gross Chronic Infarcts	32.99%	1
	Chronic Microinfarcts	29.11%	1
	Gross Infarcts	41.98%	1
	Microinfarcts	37.18%	1
Education	Education (years)	16.39 [3, 30]	0
Personality	Anxiety (range 0 to 10 [more anxious])	1.52 [0, 10]	122
	Neuroticism (range 0 to 24 [more neurotic])	8.12 [0, 18]	51
Vision	Vision (range 1 to 7 [poor])	1.55 [1, 7]	8
APOE	*APOE* 2 Allele	15.93%	13
	*APOE* 4 Allele	24.92%	13
Pain	Lower Extremity	31.7%	2
	Upper Extremity	19.73%	2
Alcohol	Lifetime (range 0 to 6 [more consumption])	0.41 [0, 6]	6
	Last 12 months (log grams per day)	0.70 [0, 4.66]	6
Smoking	Former Smoker	28.3%	5
	Current Smoker	2.56%	5
BMI	BMI	26.77 [12.65, 47.44]	29
Comorbidities	Hypertension	47.7%	1
	Cancer	32.38%	1
	Diabetes	13.48%	1
	Head Injury	5.93%	2
	Thyroid	17.47%	1
	Heart Disease	15.83%	1
	Stroke	10.43%	21
Parkinsonism	Bradykinesia (range 0 to 100 [more bradykinesia])	13.99 [0, 80]	5
	Gait Quality (range 0 to 100 [more gait problems])	19.34 [0, 100]	9
	Overall (range 0 to 2 [high])	1.07 [0, 2]	4
	Rigidity (range 0 to 100 [more rigid])	4.71 [0, 85]	4
	Tremor (range 0 to 100 [more tremor])	3.60 [0, 69.70]	5
Depression	Depression (range 0 to 10 [more depressed])	1.2 [0, 9]	5
Life Activities	Instrumental (0 to 8 [needs more help])	1.35 [0, 8]	7
	Basic (0 to 6 [needs more help])	0.23 [0, 6]	6
	Mobility Disability (0 to 3 [needs more help])	0.91 [0, 3]	8
Physical Activities	Overall (higher scores more active)	0.71 [0, 18]	4
	Walking (higher scores more active)	1.46 [0, 35]	5
	Yardwork (higher scores more active)	0.29 [0, 35]	3

### Statistical methods

We validate the AD-CR Score as a measure of CR by (1) demonstrating the score’s association with measures already known to be associated with CR and (2) demonstrating the association of the score with longitudinal global cognition trajectories. In addition, we also build a machine learning framework to predict whether an individual will have a high or low AD-CR Score using only clinical measures available during the individual’s lifetime.

#### AD-CR Score associations

We validate the proposed AD-CR Score as a measure of CR by establishing the associations of the score with measures already known to be associated with CR in the literature. Individuals with higher cognition at baseline [8], higher educational attainment [2, 8, 15], less neuroticism [44], less depressive symptoms [44, 45], less disability in daily life activities [44, 46] and more engagement in physical activities [46] have been previously found to have higher CR. Having the *APOE* E4 allele has been found to increase the risk of AD, while the *APOE* E2 allele has been shown to be protective for AD [37, 38]. Non-AD pathology [2] and other comorbidities, especially Parkinson’s disease [47], may also explain cognition that is less than expected in relation to AD related pathology. Alcohol consumption and smoking have not been found to be associated with CR [48], so we also investigate these associations to confirm the previous findings. In addition, we explore the relationship with CR and other available measures for the participants (anxiety, vision, lower and upper extremity pain, and BMI) in an effort to find new associations with the AD-CR Score and CR. To reduce the number of tests performed, we grouped these measures into 16 groups as described in the section *Other measures*: demographic, cognition, non-AD pathology, education, personality, vision, APOE, pain, alcohol, smoking, BMI, comorbidity, parkinsonism, depression, life activities, and physical activities (Table 2).

For this analysis, we first use the baseline value (collected at the participant’s first evaluation) for each of the measures as well as the non-AD pathology measures collected at autopsy. We perform a univariate nested ANOVA (or F-test) to test whether the model with the demographic group has a significantly better fit compared to the null model (in terms of the variance explained in the AD-CR Score). We then use the univariate nested ANOVA to determine if adding each of the groups from Table 2 individually fits significantly better than the demographic group model alone. Next we performed a multivariate nested ANOVA (or F-test), testing whether a model fit with all groups fits significantly better than each of the models fit after removing one of the groups. Note that the demographic group is excluded from such removal and is thus always included in all multivariate models. Additionally, as baseline cognition and pathology are highly associated with CR and many of the other measures of interest, we fit another set of multivariate nested ANOVA excluding the cognition and pathology groups from removal, which always include these two groups in addition to the demographic group in all multivariate models. Participants with missing measurements were excluded from models for which there was missingness. To adjust our inference for multiple testing, we performed multiple-comparison correction within the nested univariate ANOVA and the two nested multivariate ANOVA settings by controlling the false discovery rate at 10% [49].

Acknowledging that participants in the ROS and MAP studies were recruited into these studies at different ages, we also perform the univariate and multivariate ANOVAs using measures from participants in cross-sectional models at age 75, 80, 85, and 90, as well as the pathology measures collected at autopsy. This analysis is done in an attempt to temporally register the subjects using age. The number of participants in these groups was significantly smaller than the full study population, n = 198, 353, 413, and 284 respectively, with missingness of 1%, 2%, 3%, and 4% respectively. We therefore imputed the data with nonparametric missing value imputation using a random forest implemented in the missForest R package [50, 51]. The sensitivity analysis for this imputation can be found in the S2 Appendix. For all of the nested ANOVA models we use only sex from the demographic group; adjusting for age is not necessary as all participants in the models are the same age. Just as with the nested ANOVA models with the baseline data, we also adjust for multiple testing using the false discovery rate.

#### Association with cognitive decline

In addition to demonstrating the cross sectional associations with the AD-CR Score, we also examine the association of the AD-CR Score with the longitudinal trajectories of global cognition to provide validation for the score. Previous studies have identified a relationship between cognitive decline and CR [17]; those individuals with higher CR experience a slower rate of decline in cognition than those with lower CR, who experience a steeper drop in cognition over time. To explore the association between the AD-CR Score and cognitive decline, we consider a non-linear longitudinal mixed effects model, using splines to fit non-linear longitudinal effects [52]. In the model, we include the fixed effects of age at baseline, AD-CR Score, an interaction of age at baseline and AD-CR Score, a quadratic B-spline basis of age (henceforth referred to as the non-linear age term), and an interaction between the non-linear age term and the AD-CR Score, as well as a random effect for each subject. The baseline age and AD-CR Score fixed-effects in the model allow us to test for an overall shift in the cognition trajectory based upon age of entry into the study and CR. We include a fixed effect interaction between the AD-CR Score and the baseline age to test if the AD-CR Score modifies the relationship between the age at baseline and the longitudinal trajectories. The non-linear age term is used to capture the expected overall decline in cognition as an individual ages. In addition, we include an interaction with the AD-CR Score and this non-linear age term to test if the AD-CR Score modifies this decline. The random subject effect allows for individual differences in cognition among the subjects.

#### Prediction of the AD-CR Score

To assess the clinical utility of the AD-CR Score, we seek to predict the AD-CR Score and specifically whether subjects will have high or low AD-CR Score using only measures that are available at the baseline visit (we exclude pathology as this is only available after death). First, we randomly assign 60% of the 791 participants with complete data to a training set (490) and assign the remaining participants to a test set (301). We focus on predicting subjects that will have extreme AD-CR Scores as these are the most clinically relevant groups. Thus, we employ a two stage predictive modeling process: First, we use a predictive model to predict subjects as having extreme or average AD-CR Scores; and second, we build separate predictive models for each group to classify subjects as having high or low AD-CR Scores (defined as positive or negative AD-CR Scores respectively). For the first stage, we fit weighted regression models to predict the continuous AD-CR Score, where subjects with extreme AD-CR Scores are up-weighted. We implement regression models such as regularized regression (R package glmnet), random forests (R package randomForest), and gradient boosting (R package mboost and XGBoost). All models are trained using the training set. In particular, we use 10-fold cross-validation to choose the optimal tuning parameters, the optimal weighting scheme, as well as the best overall model using the training set. After the first stage, subjects with an absolute predicted AD-CR Score greater than 0.1 are placed into the extreme AD-CR group and the rest are placed into the average AD-CR group. For the second stage, we fit separate models to the extreme and average AD-CR groups to further classify subjects as having high or low AD-CR Scores. For this, we implement weighted classification models such as regularized logistic regression, random forests, support vector machines (Python toolbox scikit-learn), and gradient boosting; again, we use 10-fold cross-validation to choose tuning parameters and decide on the best model using the training set. The predictive performance of our machine learning framework is reported on the test set in terms of Sensitivity, Specificity, F-Score as well as accuracy for predicting whether subjects will have high or low AD-CR Scores.

## Results

### The Alzheimer’s Disease Cognitive Resilience Score

**Fig 1:**
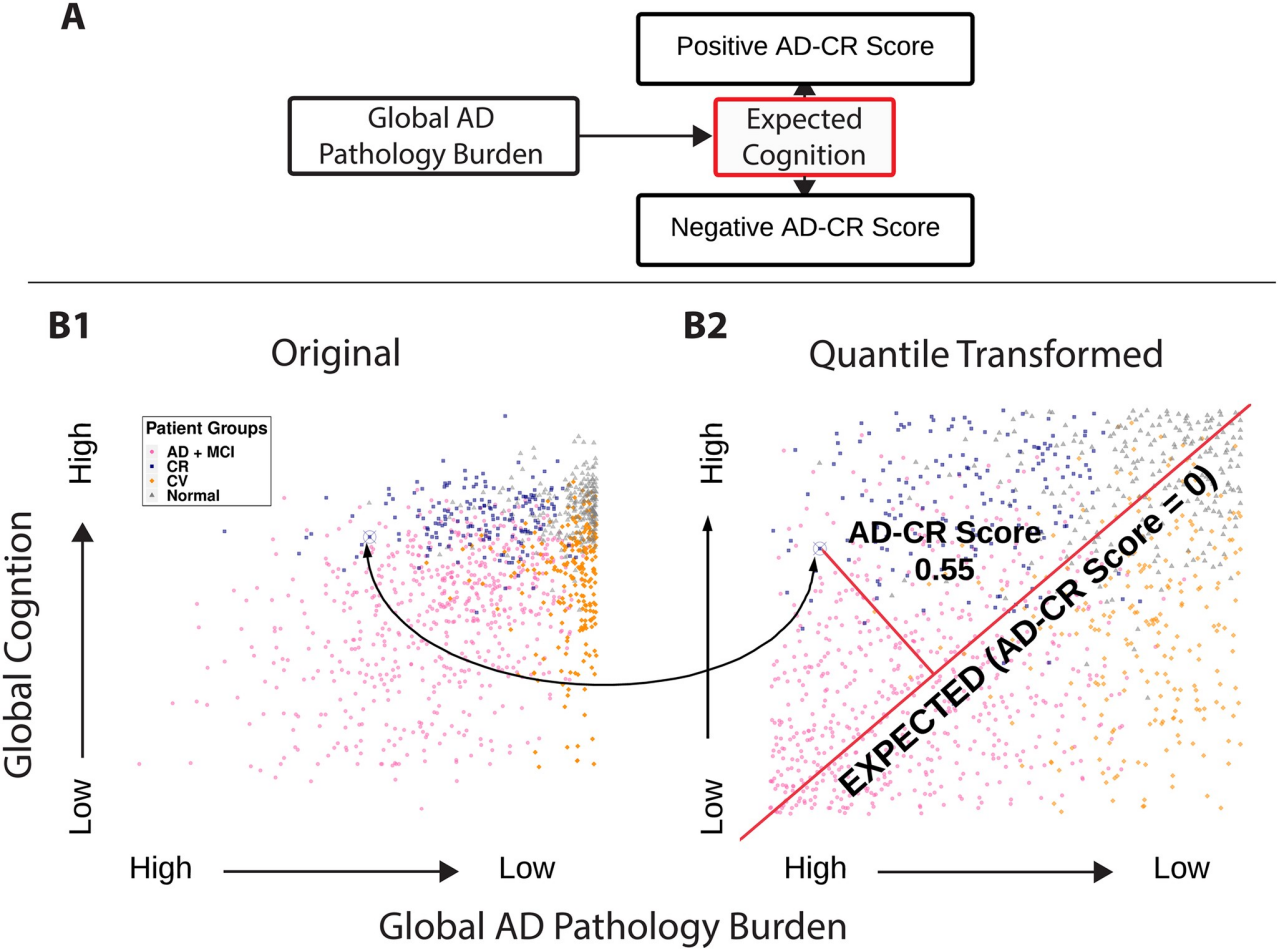
Illustrations of the AD-CR Score definition. (A) Diagram of the AD-CR Score definition. (B) Plot of the observed global cognition at last visit versus AD pathology burden of the participants where (B1) are the original values and (B2) are the quantile transformed values. A participant with a AD-CR Score of 0.55 is shown as the circled blue point. The geometric interpretation of the AD-CR Score (as the normalized shortest distance from an individual’s observed cognition and pathology to the expected values of cognition and pathology) is also shown in the plot with the red lines. Colors for the plots are determined as follows: AD and MCI (AD and MCI with high AD pathology), CR (NCI with high AD pathology), CV (AD and MCI with low AD pathology) and normal (NCI with low AD pathology).

Numerous studies have shown a link between AD-related pathology and cognitive decline [2]. Our objective is to develop a quantitative definition of CR in AD that is stand-alone, non-parametric, and produces individual quantifications independent of other measures. To this end, we first characterize the expected level of cognition given an observed level of AD pathology. The AD-CR Score is then defined as the difference between the observed and expected cognition for a given AD pathology burden. Fig 1A shows a diagram of this definition. Our AD-CR Score is computed using the global cognition measure and the global AD pathology measure (described in the *Neuropsychological performance testing* and *Neuropathologic evaluation* sections respectively).

To mathematically define our AD-CR Score, let *c* be the observed global cognition at last visit and let *p* be the observed global AD pathology. We first quantile transform the global cognition and global AD pathology: Let ${\hat{F}}_{c}$ and ${\hat{F}}_{p}$ be the empirical distribution functions for *c* and *p* respectively. The quantile transformed values of global cognition and pathology are ${\hat{F}}_{c}\left(c\right)$ and ${\hat{F}}_{p}\left(p\right)$. Fig 1B shows plots of the observed and quantile transformed global cognition at last visit versus the AD pathology for all participants in the analysis. We then define the expected level of cognition given the level of AD pathology to be the line at which the two quantiles are equal, ${\hat{F}}_{c}\left(c\right)={\hat{F}}_{p}\left(p\right)$, denoted as the red forty-five degree line in Fig 1B2. Quantile matching provides a non-parametric way to characterize the expected cognition given the level of AD pathology. Finally, we define the AD-CR Score for participant *i* with global cognition score of *c*_*i*_ and global AD pathology burden of *p*_*i*_ as:

$$\begin{array}{c} CR_{i}={\hat{F}}_{c}\left(c_{i}\right)-{\hat{F}}_{p}\left(p_{i}\right). \end{array}$$

In other words, the AD-CR Score for each individual is the difference between their quantile-transformed cognition and their quantile-transformed AD pathology level. Hence, the AD-CR Score is a continuous measure, taking values between −1 and 1. Positive values indicate that there is higher cognition than expected, negative values indicate that there is lower cognition than expected, and values near 0 indicate that the cognition is as expected for the observed pathology. Our AD-CR Score can also be interpreted as the normalized shortest distance from an individual’s observed cognition and pathology to the expected values of cognition and pathology; a proof of the mathematical equivalence of the two definitions is provided in the S1 Appendix. Fig 1B illustrates how our AD-CR Score is calculated by highlighting a participant with a AD-CR Score of 0.55 shown on both the observed and quantile transformed plots.

### Cross sectional associations

Next, we seek to validate our AD-CR Score by testing its association to previously implicated indicators of cognitive resilience in AD. Table 3 shows cross-sectional association results from the three baseline nested ANOVA models: the univariate ANOVA, the multivariate ANOVA, and the multivariate ANOVA excluding cognition and pathology. Note that we do not test the demographics group in the multivariate models as we adjust for age in all of the multivariate models. After correcting for multiple testing, we found associations with the AD-CR Score and demographic information, baseline cognition, non-AD related pathology, education, personality, *APOE* genotype, parkinsonism, depression, and life activities in the univariate ANOVA. These findings replicate previous findings in the literature [2, 8, 37, 38, 47]. In the univariate ANOVA, the only finding that we were not able to replicate from the literature was physical activity; this is perhaps due to how these measures were obtained in the ROS and MAP cohorts. In the multivariate ANOVA, cognition, non-AD pathology, and *APOE* were found to be associated with the AD-CR Score. As baseline cognition and pathology are highly associated with CR and many of the other measures of interest, we also fit a multivariate ANOVA model excluding cognition and pathology. In this model, *APOE* genotype, parkinsonism, and life activities were found to be associated with the AD-CR Score.

**Table 3:** Table of the FDR adjusted p-values from the baseline ANOVA models.

Baseline ANOVA Models (p-values)			
Group	Univariate ANOVA	Multivariate ANOVA (n = 794)	Multivariate ANOVA (n = 782) Excludes Cognition and Path
Demographics	<.001 (n = 980)	NA	NA
Cognition	<.001 (n = 974)	<.001	NA
Non-AD Pathology	<.001 (n = 969)	0.001	NA
Education	<.001 (n = 980)	0.967	0.062
Personality	<.001 (n = 858)	0.661	0.062
Vision	0.218 (n = 972)	0.967	0.406
APOE	<.001 (n = 967)	<.001	<.001
Pain	0.469 (n = 978)	0.661	0.444
Alcohol	0.074 (n = 974)	0.093	0.406
Smoking	0.218 (n = 975)	0.153	0.218
BMI	0.251 (n = 951)	0.089	0.406
Comorbidity	0.474 (n = 958)	0.967	0.737
Parkinsonism	<.001 (n = 970)	0.092	0.006
Depression	<.001 (n = 975)	0.967	0.794
Life Activities	<.001 (n = 970)	0.074	0.004
Physical Activities	0.81 (n = 974)	0.967	0.948

Statistically significant p-values are highlighted in blue. The number of participants used to fit each of the models is also reported in the table.

Table 4 shows the coefficient estimates from the baseline nested ANOVA models. Almost all of the coefficient estimates from the ANOVA regressions are in the same direction of already established associations of CR in the literature. Notably however, the *APOE* allele associations with the AD-CR Score are opposite to the well established associations between *APOE* genotype and AD risk. The *APOE* 2 allele is known to be protective for AD, while the *APOE* 4 allele increases the risk of AD [37, 38], and these alleles show consistent associations with AD pathologic burden [53, 54]. We observe that the *APOE* 2 allele is negatively associated with the AD-CR Score and the *APOE* 4 allele is positively associated with the AD-CR Score. In further investigation, the univariate regressions of the AD-CR Score on the *APOE* 2 allele and the *APOE* 4 allele maintain the same direction of the association (coefficient estimates of -0.061 and 0.121 respectively with p-values of 0.024 and < 0.001). The reason that the association for the AD-CR Score and *APOE* are in opposite directions likely relates to its strong association with AD pathologic burden, and how we have defined the AD-CR Score. A substantial body of literature supports that the association of *APOE* genotype with cognition is mediated by an increase or decrease in the level of AD pathology in the brain [27, 54]. Because *APOE* genotype impacts cognition through AD pathology and the AD-CR Score is intended to capture resilience in cognitive manifestations conditioning on a given level of AD pathology, it is not surprising that *APOE* does not show a strong positive correlation with AD-CR Score. In addition, the AD-CR Score is defined as the difference in the observed and expected cognition given the observed global AD pathology burden. Therefore, a positive AD-CR Score requires the presence of some AD pathology burden. And this is reasonable for modeling cognitive resilience because Alzheimer’s Disease cognitive resilience describes the phenomenon that healthy cognition can still exist in the presence of AD pathology. Because the *APOE* 2 likely protects against development of AD pathology in the first place, individuals with the *APOE* 2 allele are less likely to have AD pathology and therefore will not have a high positive AD-CR Score. And this agrees with the literature that *APOE* likely affects the development of pathology rather than cognitive resilience in the presence of pathology [27]. In contrast those with the *APOE* 4 allele will have more AD pathology burden and will therefore be more likely to have a positive AD-CR Score.

**Table 4:** Coefficients from the baseline ANOVA models.

Group	Measure	Univariate ANOVA	Multivariate ANOVA	Multivariate ANOVA (Excludes Cognition and Pathology)
Demographics	Age at Baseline	-0.006		
	Male	-0.064		
Cognition (z-scores, higher values for higher cognition)	Episodic Memory	0.013	0.022	
	Visuospatial Ability	0.016	0.017	
	Perceptual Speed	0.036	0.04	
	Semantic Memory	0.092	0.078	
	Working Memory	0.054	0.044	
Pathology	Hippocampal Sclerosis	-0.155	-0.136	
	Lewy Bodies	-0.084	-0.073	
	Gross Chronic Infarcts	-0.05	-0.052	
	Chronic Microinfarcts	-0.031	0.01	
	Gross Infarcts	-0.038	-0.001	
	Microinfarcts	0.004	-0.028	
Education	Education (years)	0.01	0	0.007
Personality	Anxiety (range 0 to 10 [more anxious])	-0.015	-0.005	-0.011
	Neuroticism (range 0 to 24 [more neurotic])	-0.008	-0.002	-0.006
Vision	Vision	-0.012	0.003	-0.01
APOE	*APOE* 2 Allele	-0.046	-0.047	-0.031
	*APOE* 4 Allele	0.111	0.141	0.109
Pain	Lower Extremity	0.017	0.03	0.038
	Upper Extremity	-0.039	-0.03	-0.024
Alcohol	Lifetime (range 0 to 6 [more consumption])	0.023	0.028	0.023
	Last 12 months (log grams per day)	0.003	-0.03	-0.015
Smoking	Former Smoker	0.015	-0.001	0.012
	Current Smoker	-0.11	-0.137	-0.126
BMI	BMI	-0.003	-0.005	-0.003
Comorbidities	Hypertension	0.001	0.001	0.017
	Cancer	0.043	0.017	0.044
	Diabetes	-0.007	0.012	-0.003
	Head Injury	0.04	0.03	0.028
	Thyroid	0.011	0.01	0.026
	Heart Disease	0.005	0.015	0.004
	Stroke	-0.043	0.02	0.013
Parkinsonism	Bradykinesia (range 0 to 100 [more bradykinesia])	-0.002	0	-0.001
	Gait Quality (range 0 to 100 [more gait problems])	-0.001	0.002	0.002
	Overall (range 0 to 2 [high])	-0.004	-0.01	-0.022
	Rigidity (range 0 to 100 [more rigid])	-0.004	-0.004	-0.004
	Tremor (range 0 to 100 [more tremor])	-0.001	0	0
Depression	Depression (range 0 to 10 [more depressed])	-0.024	0	-0.003
Life Activities	Instrumental(0 to 8 [needs more help])	-0.026	-0.004	-0.021
	Basic (0 to 6 [needs more help])	-0.04	-0.064	-0.068
	Mobility Disability (0 to 3 [needs more help])	0.006	0.004	0.007
Physical Activities	Overall (higher scores more active)	0.002	-0.004	-0.004
	Walking (higher scores more active)	-0.002	0.002	-0.001
	Yardwork (higher scores more active)	0.004	0.001	0.001

The coefficients highlighted in blue are those for which the ANOVA p-values are statistically significant.

**Fig 2:**
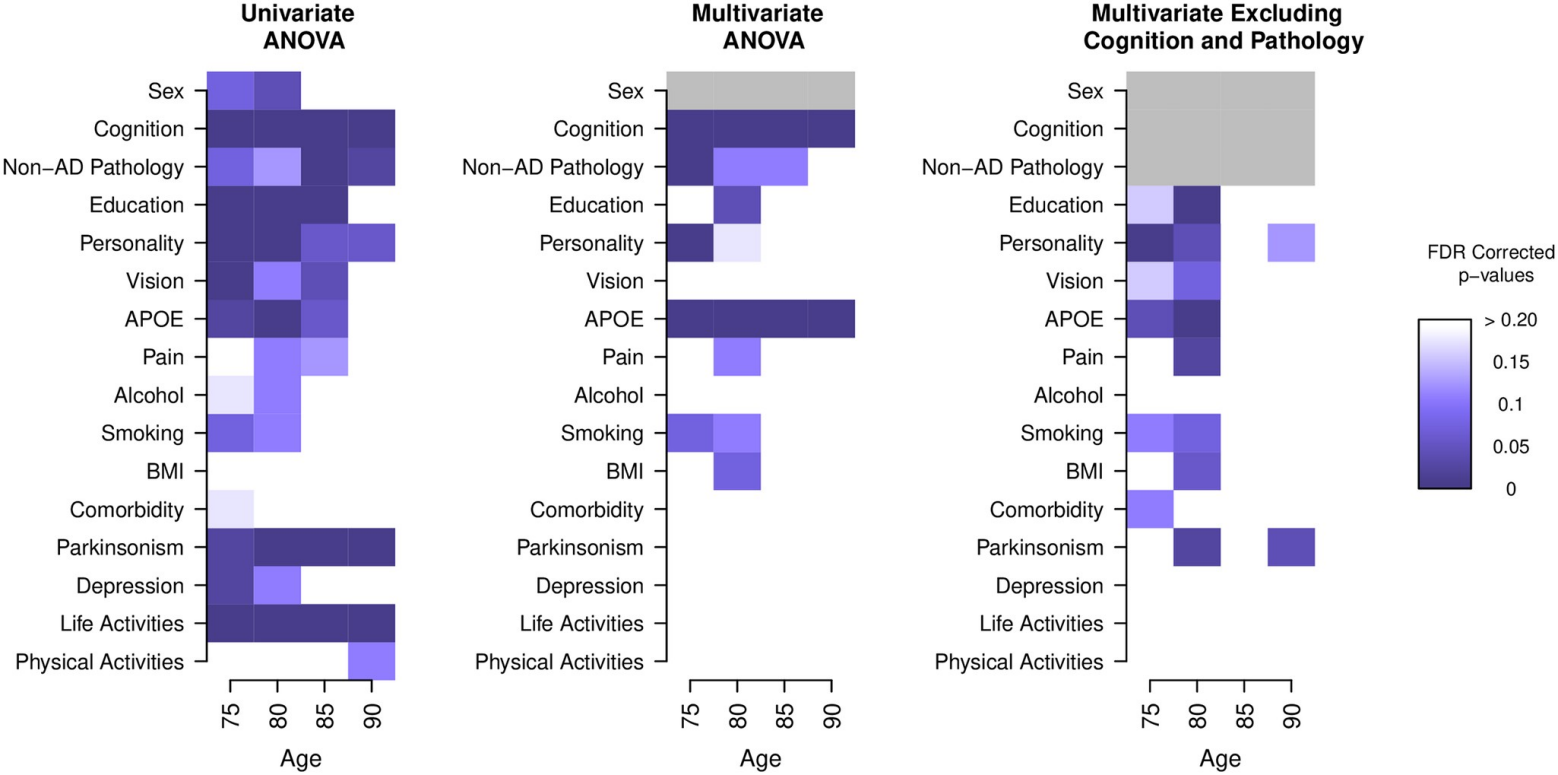
The cross-sectional nested ANOVA models for ages 75, 80, 85, and 90, with sample sizes of n = 198, 353, 413, and 284 respectively. The FDR corrected p-values are shown up to values of 0.20. We see that different measures are associated with an individual’s AD-CR Score at different time points in their lifetime and that there are more associations found in the younger age groups (75 to 80). Sex in the multivariate ANOVA model is shown in gray as all models are adjusted for sex and we do not test for an association of sex and AD-CR Score in these models. Cognition and non-AD pathology are also shown in gray as these are excluded from the multivariate ANOVA that excludes cognition and pathology.

In addition to fitting the nested ANOVA models using the baseline visit for each participant, we also fit cross sectional nested ANOVA models at age 75, 80, 85, and 90. The results from these models are shown in Fig 2 and indicate that different factors are associated with CR at different ages. Note that we do not test the sex group in the multivariate models as we adjust for sex in all of the models. An overall pattern in all of the models is that there are more significant results in the younger age groups (75 and 80) than the older groups (85 and 90). This is suggestive of the fact that there may be irreversible AD-related disease burden that cannot be modified by behaviors and other factors at these older ages. In addition, we see in the univariate model that non-AD pathology is more significant for the older groups. This indicates that non-AD pathology may account for CV, especially as participants age. The results from the cross sectional ANOVA models are similar to those in the baseline model and also validated those findings already established in the literature. In both the univariate and multivariate ANOVA models, cognition is found to be significant at all time points. The *APOE* genotype was also found to be significant at all time points in the multivariate ANOVA, and at ages 75 and 80 in the univariate and multivariate ANOVA that excluded cognition and pathology. Life activities were found to be significant at all time points in the univariate ANOVA. Non-AD pathology, education, personality, and parkinsonism were also found to be significant at different time point in the three models.

### Longitudinal cognition

**Fig 3:**
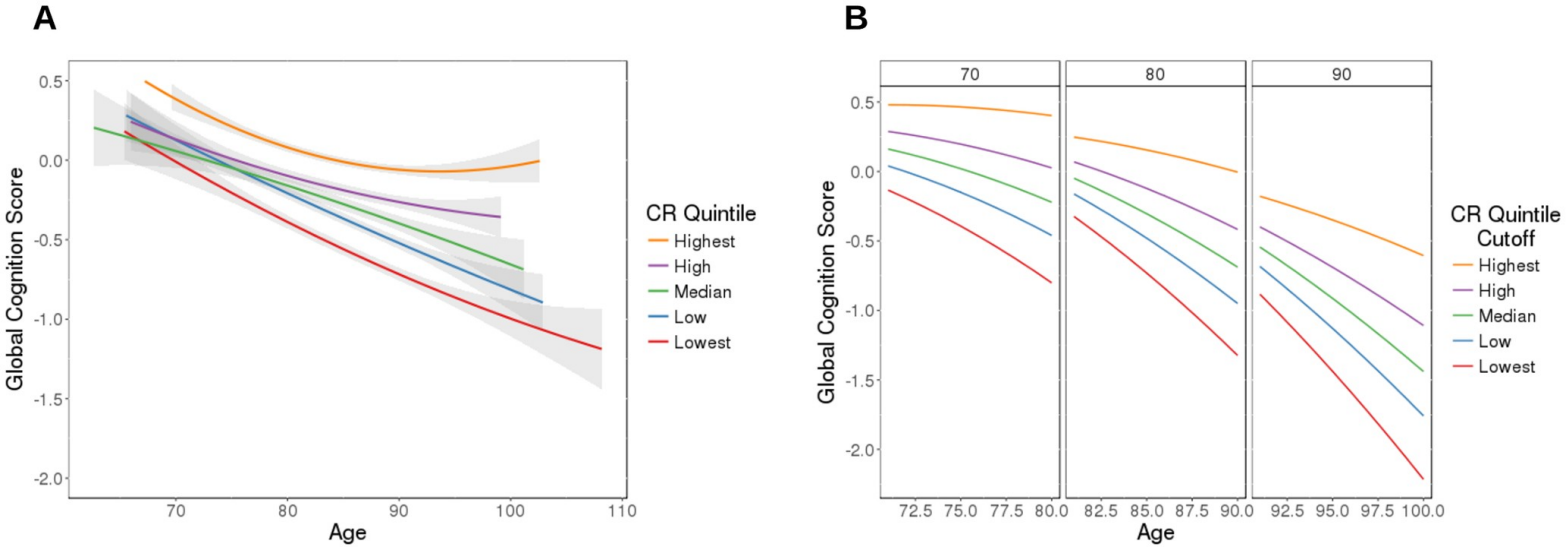
Global cognition (vertical axis) is shown over time (age, horizontal axis). (A) Longitudinal global cognition trajectories grouped by AD-CR Score quintile group. For each of the quintile groups a smoothed curve with 95% confidence band is shown. (B) The predicted longitudinal cognition trajectories for the AD-CR Score quintile groups from the fitted mixed effects model. The plot is divided into three panels, one panel each for baseline age of 70, 80, and 90 with 10 years of predictions for each of the panels.

Fig 3A shows the longitudinal global cognition for the participants versus age. The participants are grouped by AD-CR Score quintiles (Lowest, Low, Medium, High and Highest) and are smoothed using a non-linear age term within each of the quintile groups (also shown on the plot with 95% confidence bands). While all groups follow an overall decline trajectory in cognition over time, we see that participants with high AD-CR Score (those in the Highest and High quintile groups) have a much slower decline trajectory in global cognition compared to those in the lower quintile groups (those in the Low and Lowest quintile groups). This relationship has been observed in previous studies in the literature [17]; individuals with higher CR experience a slower rate of decline in cognition than those with lower CR. In addition we see that the global cognition score increases with age for those in the highest quintile of AD-CR Scores. This may be due to the fact that there are very few participants in the study who live to be over age 100; the estimates for the global cognition in these ages is less accurate than at other ages, as evident from the wider confidence bands.

In the longitudinal smoothing spline ANOVA model, all fixed effects were found to be statistically significant: baseline age (p < .001), the AD-CR Score (p < .001), the interaction between baseline age and AD-CR Score (p < .001), the non-linear age term (p < .001) and the interaction with AD-CR Score and the non-linear age term (p < .001). To illustrate the results from the model, we plot the predicted non-linear global cognitive decline trajectories from the model in Fig 3B. Each of the predicted curves in the figure represents the prediction of the cognitive trajectory for 10 years for each of the quintile group cutoffs of cognition (4 cutoffs at the quintile values). The plot is split into three panels, one panel each for baseline age of 70, 80, and 90. We note that for all quintile cutoffs, the global cognition decrease over time, and the rate of cognitive decline becomes larger with age. Participants in the higher (lower) AD-CR Score quintiles have higher (lower) overall cognition levels. Additionally we note that the rate of cognitive decline is less steep (steeper) for subjects with high (low) AD-CR Scores. Finally we note there is a discontinuity of trajectories between the three panels, demonstrating the baseline age effect. There is an increase in each of the trajectories at the start of the new panel for participants in the lower AD-CR quintile groups. This phenomenon is attributable to selection bias; subjects who enter the study at older ages have lived dementia free until the time of enrollment and are therefore healthier than the general population.

### Prediction

**Fig 4:**
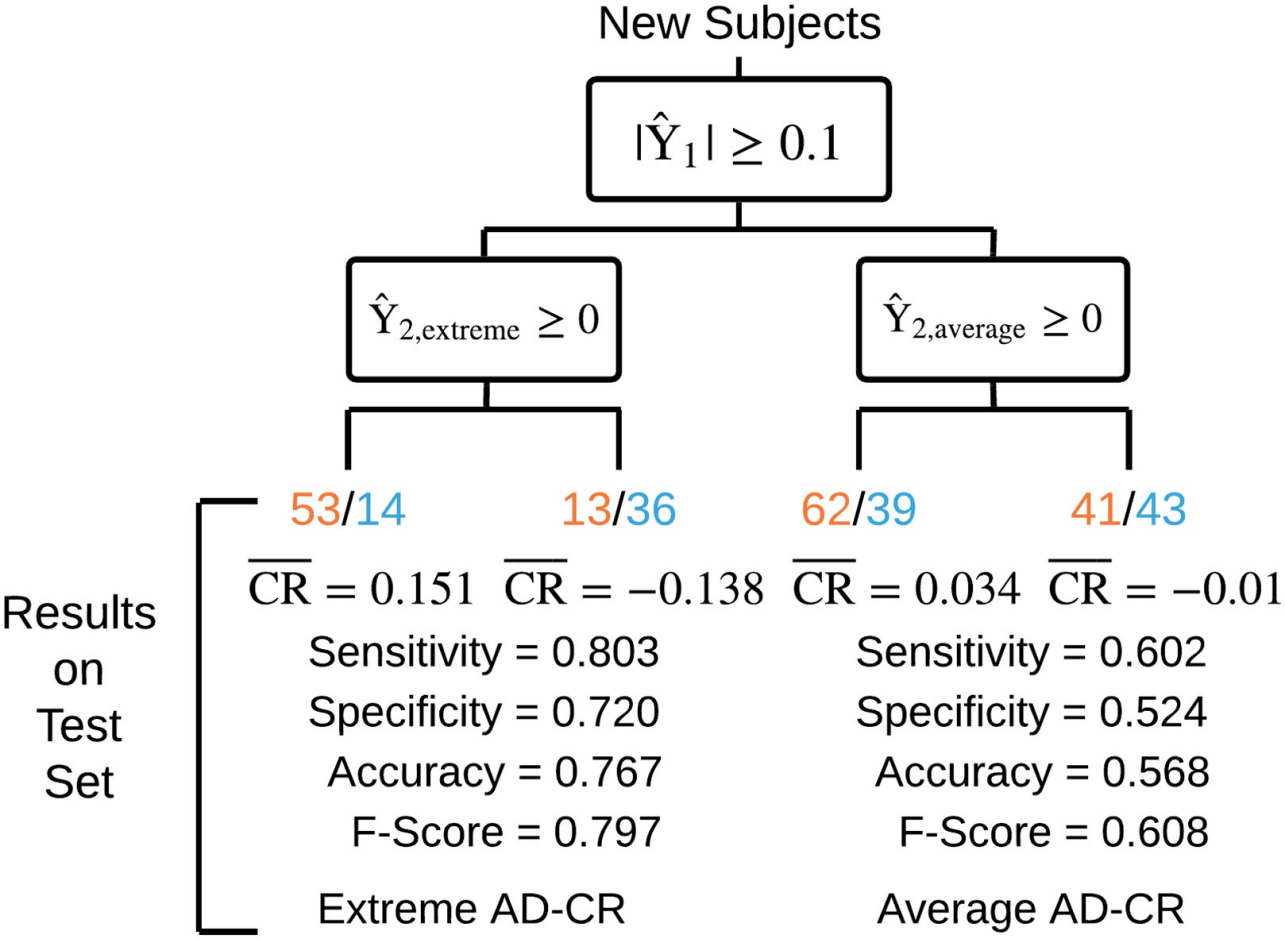
Visual depiction of our two stage machine learning framework and the predictive performance in terms of classifying high AD-CR Scores from low AD-CR Scores on the test set. The first stage model separates subjects into an Extreme AD-CR group and an Average AD-CR group based on whether their predicted AD-CR Scores are above a threshold, $|{\hat{Y}}_{1}|\geq 0.1$. Within each group separately, the second stage model classifies subjects as having either high or low AD-CR, ${\hat{Y}}_{2}\geq 0$. We report the Sensitivity, Specificity, Accuracy, and F-Score of our machine learning framework when applied to the test set and also report the number of test subjects with high (orange) or low (blue) AD-CR Scores as well as the average AD-CR Score ($\bar{CR}$) in each of the predicted categories. Overall, our machine learning framework shows promising results in terms of predicting whether an individual will have an extremely high or low AD-CR Score using only baseline measures that are available during the lifetime.

We applied our two stage machine learning framework to classify subjects as having extremely high or low AD-CR Scores, as these are the most clinically relevant groups. For these analyses, we restricted to baseline factors that are measured through clinical assessments during lifetime. Out of the machine learning models examined, cross-validation on the training set determined that gradient boosting (cross-validation-selected tuning parameters: mstop = 2000, nu = 0.1) with component-wise linear models (R package mboost) performed best in the first stage for separating subjects into an extreme AD-CR group and an average AD-CR group; additionally, cross-validation on the training set determined that linear support vector machines (SVM) were best for classifying subjects as having high or low AD-CR in stage two for both groups. Specifically, the linear SVM for the extreme AD-CR group has cross-validation-selected tuning parameter C = 0.003 and the linear SVM for the average AD-CR group has cross-validation-selected tuning parameter C = 0.0046. The predictive performance reported on the test set is visually depicted in Fig 4 and shows that our machine learning framework is able to predict whether new subjects will have extremely high (positive) AD-CR Scores vs. extremely low (negative) AD-CR Scores reasonably well (Sensitivity = 0.803, Specificity = 0.720, Accuracy = 0.767, and F-Score = 0.797 on the test set). We are not able to as accurately predict the AD-CR Scores of subjects in the average AD-CR group whose AD-CR Scores are close to zero (Sensitivity = 0.602, Specificity = 0.524, Accuracy = 0.568, and F-Score = 0.608 on the test set). Overall, our machine learning framework shows promising results in terms of predicting whether an individual will have an extremely high or low AD-CR Score using only baseline measures that are available during the lifetime.

## Discussion

CR is the mechanism that allows some individuals to be more resilient to the pathology associated with AD than others. We provide a quantitative definition of CR with the AD-CR Score; given the AD related pathology, the AD-CR Score is the difference between the observed and expected cognition. The AD-CR Score is not the first definition of CR to be proposed. Previous work has defined CR using proxy variables [2, 9–11], as a latent factors [13, 14] and using residual cognition [15–17]. Proxy variables are a poor surrogate for CR as the variables used are not standardized among individuals and are correlated with other variables known to be associated with AD. Both the latent factor and residual cognition definitions rely on parametric models, which may be misspecified. The latent factor model uses in-vivo MRI to characterize AD-related pathology in place of ‘gold standard’ postmortem pathology. Residual cognition, the definition that is closest to the AD-CR Score, uses postmortem pathology, but can only be calculated at death. In contrast to the previously proposed definitions, the AD-CR Score is a fully non-parametric, stand-alone, individual-level quantification of CR that is derived independently of other factors or proxy variables. The AD-CR Score is computed with a global measure of cognition collected proximal to death and a ‘gold standard’ AD pathology measures collected at autopsy. Unlike residual cognition methods, we also provide a framework for prediction of the AD-CR Score using information available during the lifetime.

To validate the AD-CR Score as a measure of CR, we demonstrated the association of the AD-CR Score with baseline cognition, non-AD pathology, education, personality, APOE, parkinsonism, depression and life activities in the univariate ANOVA models. These measures have previously been shown to be associated with CR in the literature [2, 8, 37, 38, 47]. Consistent with prior studies [48], we also replicated the findings that smoking and alcohol consumption are not related to CR. We were unable to replicate the association between CR and physical activity. This could be due to the fact that the available activity measures are self reported. Self reported measures of physical activity are known to be biased, especially in elderly populations such as the population in this study [55]. Recent advances in monitoring physical activity in the elderly have included the use of activity monitors to obtain a less biased measure of activity [56], which could be useful for an analysis like this. We also found that the associations with the AD-CR Score and the measures changed over time. Many of the results from the baseline ANOVA models were confirmed in the cross-sectional ANOVA models for age. We also found associations with variables that were previously not found to be associated with CR: pain in the upper and lower extremities, vision and BMI. Due to the small sample sizes for these analysis further investigation is warranted. In addition we also found associations with the AD-CR Score and longitudinal cognitive decline that were consistent with those already established in the literature. Subjects in the upper quintiles of AD-CR Score showed slower cognitive decline than those in the lower quintiles of AD-CR Score.

In addition to validating the AD-CR Score by establishing previously observed associations with CR, we also built a framework for predicting the AD-CR Score. The AD-CR Score is calculated using postmortem pathology that is only available after death. This is ideal as ‘gold standard’ postmortem pathology is the only way to definitely diagnose AD, yet it is also a limitation as the AD-CR Score cannot be calculated during an individual’s lifetime. To increase the utility of the score, we built a machine learning framework to predict the AD-CR Score using measures collected at the baseline visit. Our study shows promising results in terms of classifying extremely high AD-CR Score from extremely low AD-CR Score. Future work includes expanding the prediction model to include environmental factors, activity measures, genomic information, in-vivo brain imaging measures to further increase the predictive accuracy. Accurate prediction of the AD-CR Score would allow the score to be used in clinical practice and in clinical trial settings. If individuals are identified as having low CR using the AD-CR Score, potential interventions could increase CR and reduce their risk of cognitive decline. In clinical trials for AD disease modifying therapies, it may be useful to enrich for participants with lower CR that have the potential to show the most improvement with the therapy.

Additionally, even though we used the global cognition measure and global AD pathology measure from the ROSMAP study to compute and validate our AD-CR Score in this study, the mathematical definition for the proposed AD-CR Score is very flexible and can accommodate a variety of cognition measures as well as other AD-related pathology measures. We look to further validate our proposed AD-CR Score using other measures of cognition such as MMSE and/or other AD-related pathology in future works and evaluate whether the AD-CR Score approach works well on other populations in other studies. Furthermore, we would love to further explore the use of various smoothing techniques in future works to mitigate the potential high variability from cognitive measures in subjects with AD.

In the current form, the AD-CR Score can be used as a research tool to further understand CR and factors that are associated with CR in a quantitative manner. As demonstrated here, the AD-CR Score may be applied for discovery of epidemiological factors that are associated with and/or predictive of CR. The AD-CR Score may also be used to discover genomic and in-vivo brain imaging markers of CR. Such advances would not only enhance our understanding of the mechansims of CR in AD pathogenesis, but also has the potential to powerfully inform stratification for clinical trials of potential disease-modifying therapies for AD.

## Supporting information

S1 Appendix Geometric definition of the AD-CR Score. (PDF)

S2 Appendix Sensitivity analysis for cross sectional age ANOVA. (PDF)
