# Supplementary material for: Quantifying cognitive resilience in Alzheimer’s Disease: The Alzheimer’s Disease Cognitive Resilience Score
Source: PLoS One. 2020 Nov 5;15(11):e0241707. doi: 10.1371/journal.pone.0241707 (PMC7643963; doi:10.1371/journal.pone.0241707)
Supplement: S1 Appendix — (PDF) [file pone.0241707.s001.pdf]

S1 Appendix: Geometric Definition of the AD-CR Score

The AD-CR Score can also be defined as the smallest (signed) Euclidean distance from the observed pair of global cognition and pathology to an expected pair of global cognition and pathology (those which satisfy $\hat{F}_c(c) = \hat{F}_p(p)$). For a participant i with global cognition score of c_i and global pathology score of p_i, this is the shortest distance of $\hat{F}(c_i)$ and $\hat{F}(p_i)$ from $\hat{F}_c(c) = \hat{F}_p(p)$, expressed as $\frac{|\hat{F}_c(c) - \hat{F}_p(c)|}{\sqrt{2}}$. We remove the absolute value, which indicates whether the transformed cognition is greater than the transformed pathology, and then multiplied by $\frac{2}{\sqrt{2}}$ to normalize the score so that it takes values between -1 and 1. This gives us the $\hat{F}_c(c_i) - \hat{F}_p(p_i)$, which is the same as the definition of the AD-CR Score provided in the subsection *The Alzheimer's Disease Cognitive Resilience Score* :

$$CR_i = \hat{F}_c(c_i) - \hat{F}_p(p_i)$$
