# Supplementary material for: Quantifying cognitive resilience in Alzheimer’s Disease: The Alzheimer’s Disease Cognitive Resilience Score
Source: PLoS One. 2020 Nov 5;15(11):e0241707. doi: 10.1371/journal.pone.0241707 (PMC7643963; doi:10.1371/journal.pone.0241707)
Supplement: S2 Appendix — (PDF) [file pone.0241707.s002.pdf]

S2 Appendix: Sensitivity Analysis for Cross Sectional Age ANOVA

For our sensitivity analysis, we use the cross-sectional data for participants at age 85. Of the 353 participants with observations at age 85, 3% of the measurements are missing with only 260 participants having complete data. For our sensitivity analysis, we use the 260 patients with complete data and simulate 3% missingness at random over all of the complete data. We then use the missForest R package [1, 2] to compute the missing values. We repeat this procedure 500 times. To evaluate the performance of the missForest imputations, we examine the distribution of the p-values for the univariate ANOVA. Fig 1 shows the histograms of the p-values from each of the groups from the univariate ANOVA with the imputed data. The red line on each of the histograms indicates the 'true' p-values that was fit with the complete data. The distribution of the p-values is centered around the true p-value and the spread of the distribution is not very wide (especially on those measures that were found to be statistically significant in the age models).

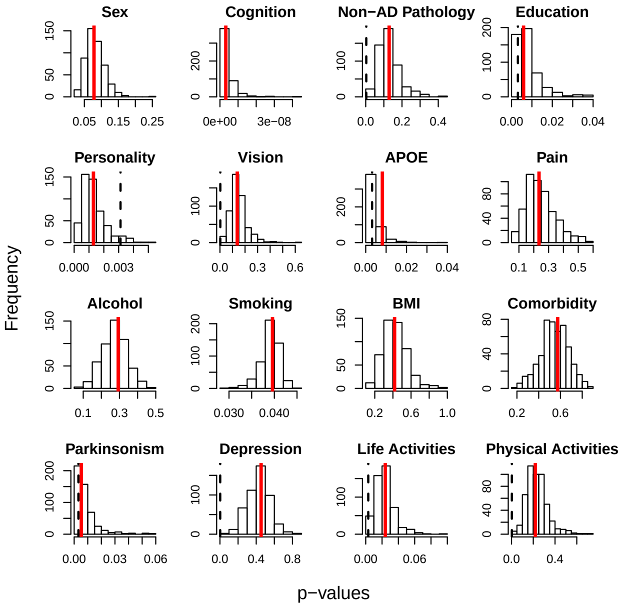

Fig 1. The histograms of p-values for each of the groups for the univariate ANOVA with the imputed data using missForest. The red line on each of the histograms indicates the 'true' p-values that was fit with the complete data. The distribution of the p-values is centered around that of the 'true' p-values.

References

1. Stekhoven DJ. missForest: Nonparametric Missing Value Imputation using Random Forest; 2013.

2. Stekhoven DJ, Buhlmann P. MissForest - non-parametric missing value imputation for mixed-type data. Bioinformatics. 2012;28(1):112–118.
